# Epidemiological and immunological insights into respiratory infections in post-COVID-19

**DOI:** 10.3389/fcimb.2025.1634415

**Published:** 2026-01-05

**Authors:** Ziyi Wang, Yi Li, Jianpiao Cai, Yujie Sun, Mulatijiang Maimaiti, Yiwei Liu, Chenglin Yang, Wenjing Liu, Sabrina Li, Yi Ren, Yu Chen, Qiwen Yang, Yingchun Xu, Jie Yi

**Affiliations:** 1 Department of Clinical Laboratory, Peking Union Medical College Hospital, Chinese Academy of Medical Sciences & Peking Union Medical College, Beijing, China; 2 State Key Laboratory for Emerging Infectious Diseases, Carol Yu Centre for Infection, Department of Microbiology, School of Clinical Medicine, Li Ka Shing Faculty of Medicine, The University of Hong Kong, Hong Kong, Hong Kong SAR, China; 3 Coyote Bioscience Research Institute, Beijing, China

**Keywords:** COVID-19, respiratory pathogens, epidemiological feature, coinfection, immuneresponses

## Abstract

**Introduction:**

Post-COVID-19 respiratory infection dynamics require updated epidemiological characterization to inform clinical surveillance and public health strategy.

**Methods:**

We analyzed 2484 patients with respiratory tract infections (September 2023–February 2024) using comprehensive pathogen screening (29 viral, bacterial, and atypical targets) and cytokine quantification (12 cytokines).

**Results:**

Overall pathogen detection was 70.73%, with viral and bacterial identification in 40.42%(1004/2484), 51.45%(1278/2484) of cases respectively, and co-infections in 31.88% (predominantly *Haemophilus influenzae*-virus). Pediatric patients (<18 years) showed significantly higher positivity (74.1% vs. 63.2%, *P* < 0.05) with viral predominance (41.57% vs. 37.84%), while adults showed bacterial predominance (57.38% vs. 38.23%). Pneumonia risk exhibited age-pathogen specificity: *Mycoplasma pneumoniae* posed the highest risk in children (41.1% pneumonia rate) versus *influenza B* in adults (10.2% detection rate). Retrospective cytokine analysis (pre-pandemic 2018–2019 vs. post-pandemic 2023–2024) revealed post-pandemic suppression of IL-6 (6.12 vs.3.82 pg/mL) and IL-8 (37.98 vs. 18.35 pg/mL), with resurgence in 2024, particularly in pediatric and pneumonia cases (P<0.05).

**Discussion:**

Post-pandemic respiratory pathogen epidemiology is characterized by heightened pediatric susceptibility to viral co-infections, bacterial pathogen persistence despite control measures, and dysregulated inflammatory responses. These findings warrant age-stratified diagnostic and surveillance approaches with adaptive public health strategies to reduce respiratory infection morbidity.

## Introduction

Coronavirus disease (COVID-19), caused by severe acute respiratory syndrome coronavirus-2 (SARS-CoV-2), is a respiratory illness primarily affecting the lungs and frequently leading to severe complications including acute respiratory distress syndrome, multi-organ failure, septic shock, and mortality (Haudebourg et al., 2020; Wang et al., 2020; Gu and Cao, 2021). The pandemic has exerted profound global impacts, disrupting public health systems, socioeconomic structures, and daily life through widespread implementation of lockdowns, travel restrictions, and business closures (Health, 2020). As societies transition toward coexisting with the virus, understanding the long-term consequences of pandemic control measures on respiratory pathogen epidemiology and immune function has become imperative (Bedford et al., 2020).

COVID-19 has substantially modified global trends in respiratory infections. Beyond introducing a novel pathogen, the pandemic has fundamentally altered the epidemiology of existing respiratory diseases. Seasonal respiratory viruses such as *influenza* and *respiratory syncytial virus* (RSV) exhibited marked fluctuations in incidence during pre-pandemic, lockdown, and post-lockdown phases, as demonstrated by longitudinal surveillance in Turkey (Kara et al., 2024). Concurrently, the pandemic influenced outcomes of chronic respiratory conditions; while *influenza* incidence declined temporarily, tuberculosis-related mortality increased due to healthcare access barriers, highlighting the complex interplay between SARS-CoV-2 and other respiratory pathogens (Jayaraman et al., 2024).

The concept of “immunity debt” has emerged as a critical concern in assessing the pandemic’s immunological legacy. This phenomenon, hypothesized to result from reduced microbial exposure during prolonged non-pharmaceutical interventions (NPIs), may increase population susceptibility to infections upon resumption of normal activities. Severe COVID-19 is characterized by dysregulated inflammatory responses, where host immune mechanisms contribute to tissue damage rather than pathogen clearance (Merad et al., 2021). These immunological alterations may have prolonged clinical implications, as evidenced by persistent T-cell activation lasting up to 12 months post-infection and delayed immune recovery in severe cases (Taeschler et al., 2022). Challenges in maintaining routine vaccination programs, exemplified by disrupted COVID-19 vaccine rollouts in regions such as Fiji, further compound risks of attenuated population immunity (Chand, 2021). Additionally, pandemic-induced financial strain on healthcare systems may indirectly exacerbate disease burdens through constrained diagnostic and therapeutic resources (Guttman-Kenney et al., 2022; Memon et al., 2022).

To address these emerging challenges, we conducted comprehensive surveillance of respiratory pathogens and cytokine profiles in Beijing during the 2023–2024 autumn-winter seasons. This study aimed to characterize post-pandemic shifts in respiratory infection patterns and evaluate associated immune perturbations through two complementary approaches: (1) large-scale screening of 29 respiratory pathogens across 2484 symptomatic patients, and (2) comparative analysis of cytokine levels in historical (2018-2019) and contemporary (2023-2024) cohorts.

## Materials and methods

### Patient enrollment

The case numbers were collected according to the study aims (**Supplementary Table S1**). All cases (aged 1 month–94 years) were diagnosed with acute respiratory infections (ARIs) or pneumonia refer to the previous study (Li et al., 2021).To be specific, for comprehensive respiratory pathogen panel testing, we included 2484 patients presenting with respiratory tract infection (RTI) symptoms to the emergency department of Peking Union Medical College Hospital (PUMCH) (during September 2023–February 2024). Nasopharyngeal swab (NPS) specimens were collected from all participants and transferred to the clinical laboratory for comprehensive respiratory pathogen panel testing before initiating therapeutic interventions.

For cytokine profiling analysis, we retrospectively examined 209 RTI cases with available biospecimens collected during three distinct epidemiological periods: November 2018–December 2019 (Group 1,n=41), September 2023–February 2024 (Group 2,n=70), and September 2024–December 2024 (Group 3,n=98). All specimens were immediately processed within 24 hours of collection following standardized protocols. The study protocol received ethical approval from the PUMCH Ethics Committee (No. I-22PJ860).

### Nucleic acid extraction and purification

The NPS were used for nucleic acid extraction by using the Nucleic Acid Extraction and Purification Kit (Xi ‘an Tianlong Technology Co., Ltd, Xi’an, China) on GeneRotex 96 Automatic Nucleic Acid Extraction instrument (Xi ‘an Tianlong Technology Co., Ltd, Xi’an, China) following producer’s protocols.

### PCR amplification

The detection of respiratory pathogens was performed using the Respiratory Tract Pathogen Nucleic Acid Detection Kit (Coyote Bioscience, Beijing, China), for the qualitative detection of nucleic acids from 29 respiratory pathogens, including 14 types of RNA viruses (*Influenza A virus* (IFVA), *Influenza B virus* (IFVB), *Parainfluenza virus type 1* (HPIV1), *Parainfluenza virus type 2* (HPIV2), *Parainfluenza virus type 3*(HPIV3), *Parainfluenza virus type 4* (HPIV4), *Coronavirus 229E*(HCoV229E), *Coronavirus OC43* (HCoVOC43), *Coronavirus NL63*(HCoVNL63), *Coronavirus HKU1*(HCoVHKU1), *Respiratory syncytial virus* (RSV), *Human metapneumovirus* (HMPV), *Human Rhinovirus* (HRV), *Measles virus* (MeV), 2 types of DNA viruses (*Human Adenovirus* (HAdV), *Human bocavirus* (HBoV)), 2 types of atypical pathogens (*Mycoplasma pneumoniae*, *Chlamydia pneumoniae*), and 11 types of bacterial species (*Group A streptococcus* (GAS)*, Streptococcus pneumoniae, Haemophilus influenzae, Legionella pneumophila, Klebsiella pneumoniae, Pseudomonas aeruginosa, Staphylococcus aureus, Moraxella catarrhalis, Escherichia coli, Acinetobacter baumannii, Bordetella pertussis* from nasopharyngeal and oropharyngeal swab samples. The detection process utilizes PCR amplification combined with fluorescence probe technology. Specific primers and fluorescent probes (FAM, VIC, Cy5, Texas Red) targeted microbial genetic material in eight separate reaction wells. Real-time fluorescence signals were recorded during amplification on Tianlong Gentier 96E. For amplification, 7µL nucleic acid extraction was added into an 18 µL prepared PCR reaction mix. The PCR cycling conditions included an initial reverse transcription step at 42°C for 5 minutes, followed by denaturation at 95°C for 1 minute, and 45 cycles of amplification (95°C for 5 seconds and 60°C for 30 seconds, with fluorescence collection). Positive and negative controls were included in each run to ensure assay validity. Data were analyzed by monitoring the threshold cycle (Ct) values, with results interpreted according to predefined quality control parameters.

### Detection of multiple cytokines

Cytokine levels were measured using multiple cytokines (12-items) Detection Kit (Flowcytometry Fluorescence Luminance Method, Joinstar Biomedical Technology Co,.Ltd., Hangzhou, China), which is based on double antibody sandwich flow cytometry and liquid suspension chip technology to detect a variety of cytokines. The 12 cytokines included interleukin (IL)-1β, IL-2, IL-4, IL-5, IL-6, IL-8, IL-10, IL-12p70, IL-17, tumor necrosis factor (TNF)-α,interferon (IFN)-α, and IFN-γ. 200µL NPS samples were measured using the kit following producer’s protocols. The fluorescent antibody signal is captured, decoded and clustered by iMatrix 100 system. According to the intensity of the fluorescent signal, the concentration of each cytokine in the sample is calculated through the calibration curve.

### Statistical analysis

Descriptive statistics for continuous variables are presented as medians with interquartile ranges (IQR). The Mann–Whitney U-test was used for comparisons between two groups. Categorical variables were expressed as % (m/n) and examined using χ2/Fisher’s exact test. *P* value < 0.05 was considered statistically significant. Statistical analyses were performed and graphs were plotted using R (4.2.1).

## Results

### Demographic characteristics

This study encompassed 2484 cases with a relatively balanced sex distribution (48.11% male, 51.89% female). The cohort was predominantly pediatric, with 69.04% (n=1715) of cases in children (<18 years) and 30.96% (n=769) in adults, reflecting a clear majority of pediatric cases during the surveillance period. Cases were temporally distributed across six months (September 2023–February 2024), with peak incidence in October (36.07%) and December (24.19%). Overall, 85.02% of cases presented as ARI, while 14.98% were diagnosed with pneumonia (**Table 1**). Characteristics of patients involved in this study were presented in **Supplementary Table S2**.

**Table 1:** Positive detection ratios of patients in this study.

Characteristic	Positive detection rate (%) (N=2484)	*P* value	Pneumonia (%) (N=2484)	Non-Pneumonia (%) (N=2484)	*P* Value
All	70.73 (1757/2484)		14.98 (372/2484)	85.02 (2112/2484)	
Gender					
Male	72.47 (866/1195)	***P*<0.05**	15.56 (186/1195)	84.44 (1009/1195)	***P***>**0.05**
Female	69.12 (891/1289)		14.43 (186/1289)	85.57 (1103/1289)	
Age (years old)					
Children	74.11 (1271/1715)	***P*<0.05**	10.79 (185/1715)	89.21 (1530/1715)	***P*<0.05**
<1	71.9 (110/153)		9.15 (14/153)	90.85 (139/153)	
1-3	68.49 (150/219)		6.85 (15/219)	93.15 (204/219)	
4-6	73.32 (316/431)		8.82 (38/431)	91.18 (393/431)	
7-12	76.61 (583/761)		11.83 (90/761)	88.17 (671/761)	
13-18	74.17 (112/151)		18.54 (28/151)	81.46 (123/151)	
Adults	63.20 (486/769)		24.32 (187/769)	75.68 (582/769)	
19-35	65.38 (187/286)		12.24 (35/286)	87.76 (251/286)	
36-60	65.88 (222/337)		22.26 (75/337)	77.74 (262/337)	
>60	52.74 (77/146)		52.74 (77/146)	47.26 (69/146)	
Months					
September, 2023	54.79 (103/188)	***P*<0.05**	26.06 (49/188)	73.04 (139/188)	***P*<0.05**
October,2023	70.31 (630/896)		12.28 (110/896)	87.72 (786/896)	
November,2023	75.69 (218/288)		11.46 (33/288)	88.54 (255/288)	
December,2023	73.88 (444/601)		14.81 (89/601)	75.2 (512/601)	
January,2024	74.14 (258/348)		16.95 (59/348)	83.05 (289/348)	
February,2024	63.8 (104/163)		19.63 (32/163)	80.37 (131/163)	

***P-*values** were calculated by Mann-Whitney U-test and χ2 test.

### Test positive rate of different pathogens

Among 2484 patients screened for 29 respiratory pathogens, 70.73%(1757/2484) tested positive for at least one pathogen 2894 pathogens were detected in 2484 cases with viral, bacterial and atypical pathogens were identified in 40.42%(1004/2484), 51.45%(1278/2484)cases, respectively (**Supplementary Table S3**). A statistically significant age-related difference was observed, with pediatric cases demonstrating higher overall positivity rates (74.11%, 1271/1715) compared to adults (63.20%, 486/769) (**Table 1**, P<0.05) This disparity was evident across both viral (41.57% vs. 37.84%) and non-viral pathogens (57.38% vs. 38.23%) in children versus adults, respectively (*P* < 0.05, **Supplementary Table S3**).

**Figure 1:**
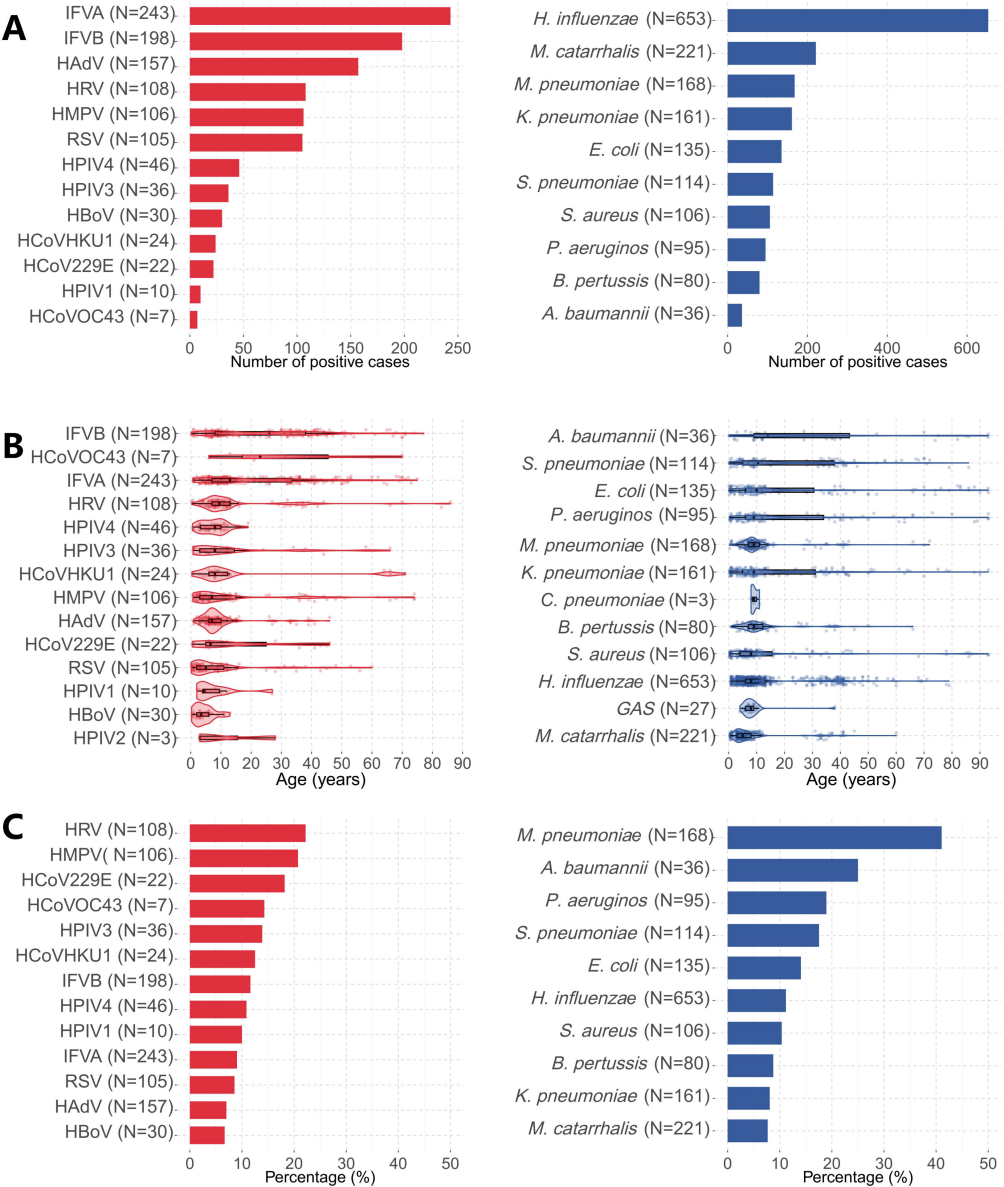
Positive number, median age and pneumonia risk of different pathogens. **(A)** Positive number for viruses on the left and bacteria on the right. **(B)** Age distribution of infections, with virus cases shown on the left and bacterial cases on the right. **(C)** Risk of pneumonia from different pathogens, Percentage data for viruses is shown on the left, and for bacteria on the right.

The predominant viral pathogens during the observation period were IFVA (9.78%, 243/2484), IFVB(7.97%, 198/2484), and HAdV(6.32%, 157/2484). Among bacterial pathogens, *Haemophilus influenzae* (26.28%, 653/2484) and *Moraxella catarrhalis* (8.90%, 221/2484) demonstrated highest prevalence, while *Mycoplasma pneumoniae* (6.76%, 168/2484) predominated among atypical pathogens. Notably, none of the 2484 cases tested positive for the three specific pathogens, HCoVNL63, MeV, and *L. pneumophila* (**Figure 1A**).

**Figure 2:**
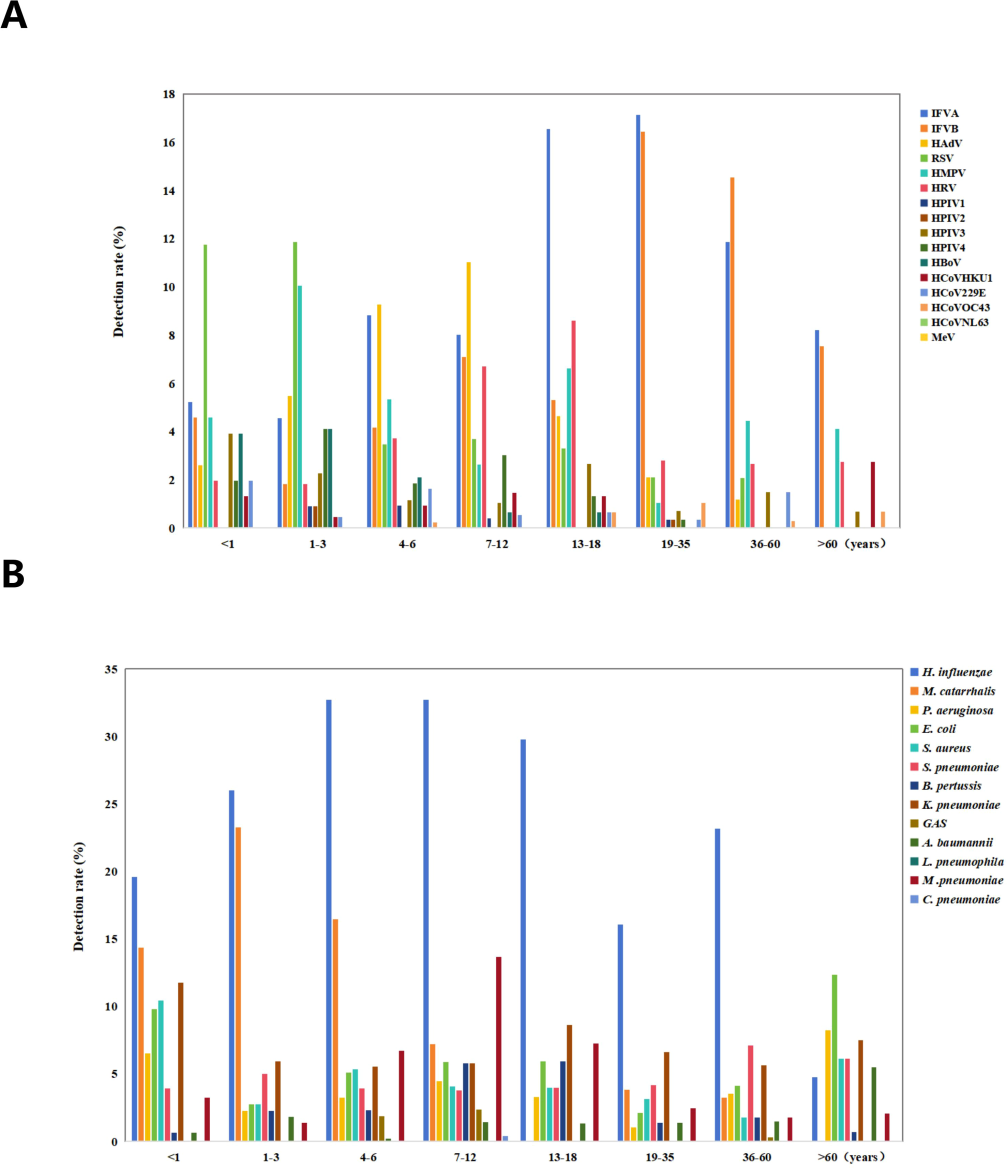
Test positive rate of pathogens in different age group. **(A)** The positive rate of the virus among different age groups. **(B)** The positive rate of bacterial infection among different age groups.

Age-specific analysis showed peak positivity among school-aged children (7–12 years: 76.61%, 583/761) and lowest detection rates in older adults (>60 years: 52.74%, 77/146) (**Table 1**). Median age of infection for most pathogens clustered around 10 years, with notable exceptions being IFVB (median age 26 years, IQR 8-38) and HCoVOC43 (median age 23 years, IQR 17-45.5) (**Figure 1B**). Age-stratified analysis of 29 respiratory pathogens demonstrated significant variations in detection patterns: both IFVA and IFVB demonstrated consistently high detection rates across all age cohorts, with particularly pronounced prevalence among youths (13–18 years)and adults (19–35 and 36–60 years). Pediatric populations exhibited significantly higher infection rates for HPIV(primarily HPIV3 and HPIV4 subtypes) compared to adult groups (**Figure 2A**). HMPV demonstrated a bimodal distribution across age extremes, with elevated rates in both pediatric and elderly cohorts. Regarding bacterial and atypical pathogens, *H. influenzae* maintained high prevalence across all age groups except seniors (>60 years), while *M. catarrhalis* showed predilection for younger pediatric populations. Notably, *M. pneumoniae* maintained broad age distribution with significant prevalence among school-aged children (**Figure 2B**).

### Pneumonia risk of different pathogens

Pneumonia complications occurred in 372 cases (14.98%,372/2484), with significant age-related disparity: pediatric patients (<18 years) exhibited lower incidence (10.79%, 185/1715) compared to adults (24.32%, 187/769; p<0.05) (**Table 1**). Pathogen-specific pneumonia risk stratification (**Figure 1C**) identified *M. pneumoniae* as having strongest association (41.1%, 69/168), followed by *A. baumannii* (25.0%, 9/36), HRV (22.2%, 24/108), and HMPV (20.8%, 22/106). Within the pneumonia group, a statistically significant disparity in viral versus non-viral pathogen detection rates was identified in pediatric patients (*P* < 0.05)(**Supplementary Table S3**).

Bacterial and viral etiologies exhibited distinct age-specific distribution patterns in pneumonia patients (**Supplementary Table S4**). *M. pneumoniae* was identified as the predominant pathogen in pediatric pneumonia cases, accounting for 33.5% of identified pathogens, whereas *H. influenzae* demonstrated higher prevalence in adult populations, constituting 12.3% of cases. Respiratory virus detection revealed significant age-related variations: HRV showed greater frequency in pediatric cases (10.3%) compared to adult cases (2.7%). Conversely, IFVB exhibited an inverse distribution pattern, being detected in 10.2% of adult patients versus 2.2% of pediatric cases.

### Co-infection patterns of respiratory pathogens

Among the cohort, 38.85% (965/2484) tested positive for a single pathogen, 31.88%(792/2484) for multiple pathogens(**Supplementary Table S5**, P<0.05). Pediatric populations demonstrated significantly higher co-infection prevalence compared to adults (37.26% vs. 19.90%, *P* < 0.05), with peak co-infection rates observed in school-aged children (7–18 years: 37.39%, 341/912) compared to the lowest rates in elderly adults (>60 years: 17.81%, 26/146) (**Supplementary Table S5**).

**Figure 3:**
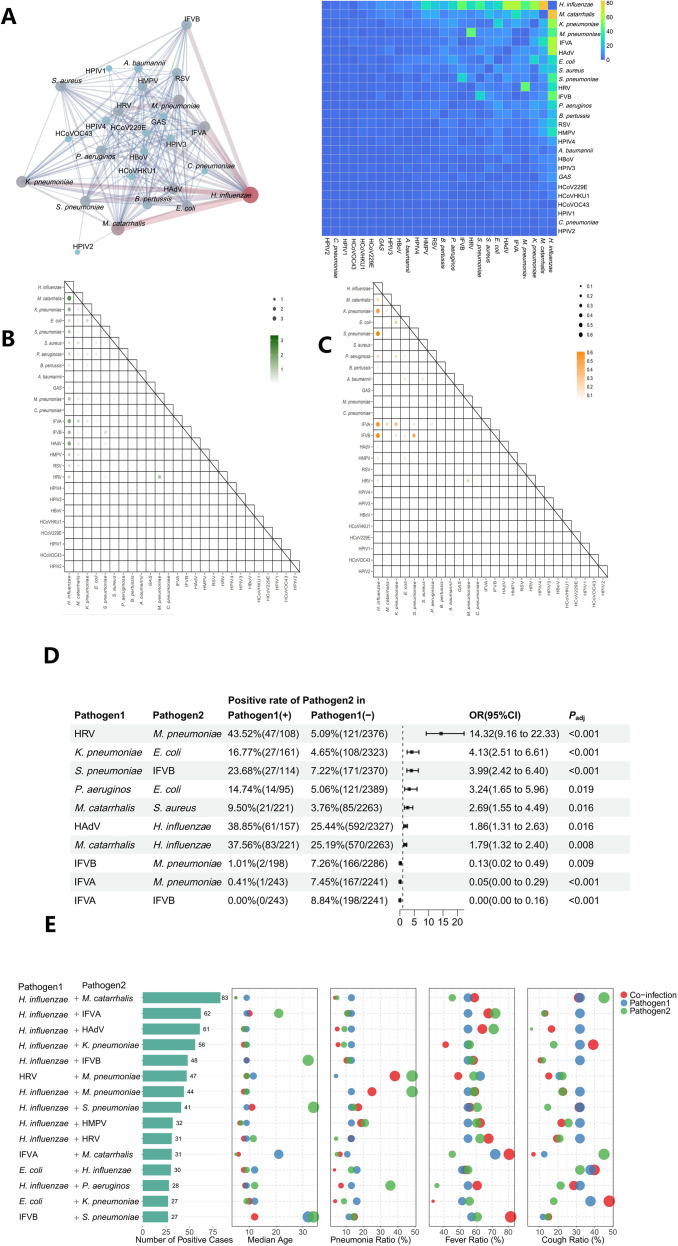
Coinfection pattern and interactions of pathogens in patients with ARIs and pneumonia in Beijing from 2023 September to 2024 February. Coinfection rates were calculated pairwise. For pathogen ‘X’ and ‘Y’, numerator was the number of patients coinfected both ‘X’ and ‘Y’ and the denominator where the total number of patients who were both tested ‘X’ and ‘Y’. **(A)** Interaction network analysis of pathogens; **(B)** Children’s pattern; **(C)** Adults’ pattern; **(D)** Pathogen combinations with significant interaction; **(E)** High-frequency combinations of co-occurrence patterns and their clinical implications.

Network analysis revealed distinct age-specific co-infection patterns. *H. influenzae* occupied a central hub in the interaction network, with 66.0% (431/653)of its cases involving co-infections.(**Figure 3A**). Pediatric cases predominantly featured *H. influenzae* with HAdV, IFVA or *M. pneumoniae*, while adult cases showed higher prevalence of *H. influenzae*-IFVB, *H. influenzae*-IFVA and *S. pneumoniae*-IFVB combinations (**Figures 3B, C**). Several pathogen pairs demonstrated statistically significant correlations: *M. pneumoniae* significantly co-occurred with HRV (43.52% co-occurrence vs. 5.09% without HRV; *P* < 0.001), while *K. pneumoniae*-*E. coli* and *S. pneumoniae*-IFVB combinations also showed significant associations (*P* < 0.05, **Figure 3D**). High-frequency co-infection combinations (prevalence >1%) peaked in children around 10 years of age (**Figure 3E**).

While co-infection status did not elevate pneumonia risk overall, specific combinations significantly increased fever incidence. Co-infection with IFVB and *S. pneumoniae* (median age 12 years) resulted in markedly elevated fever ratio (81.5%) compared to single infections with IFVB alone (57.3%, median age 32 years) or *S. pneumoniae* alone (60.7%, median age 34 years; *P* < 0.05). Similar trends were observed for IFVA and *M. catarrhali*s co-infections.

### Pattern of month-specific positivity rates

Temporal trends revealed fluctuating testing positive rate(TPR) throughout the surveillance period, commencing at 54.79% (103/188) in September 2023. Positivity rates escalated to >70% during October 2023–January 2024, before decreasing to 63.8% (104/163) by February 2024 (**Table 1**).

**Figure 4:**
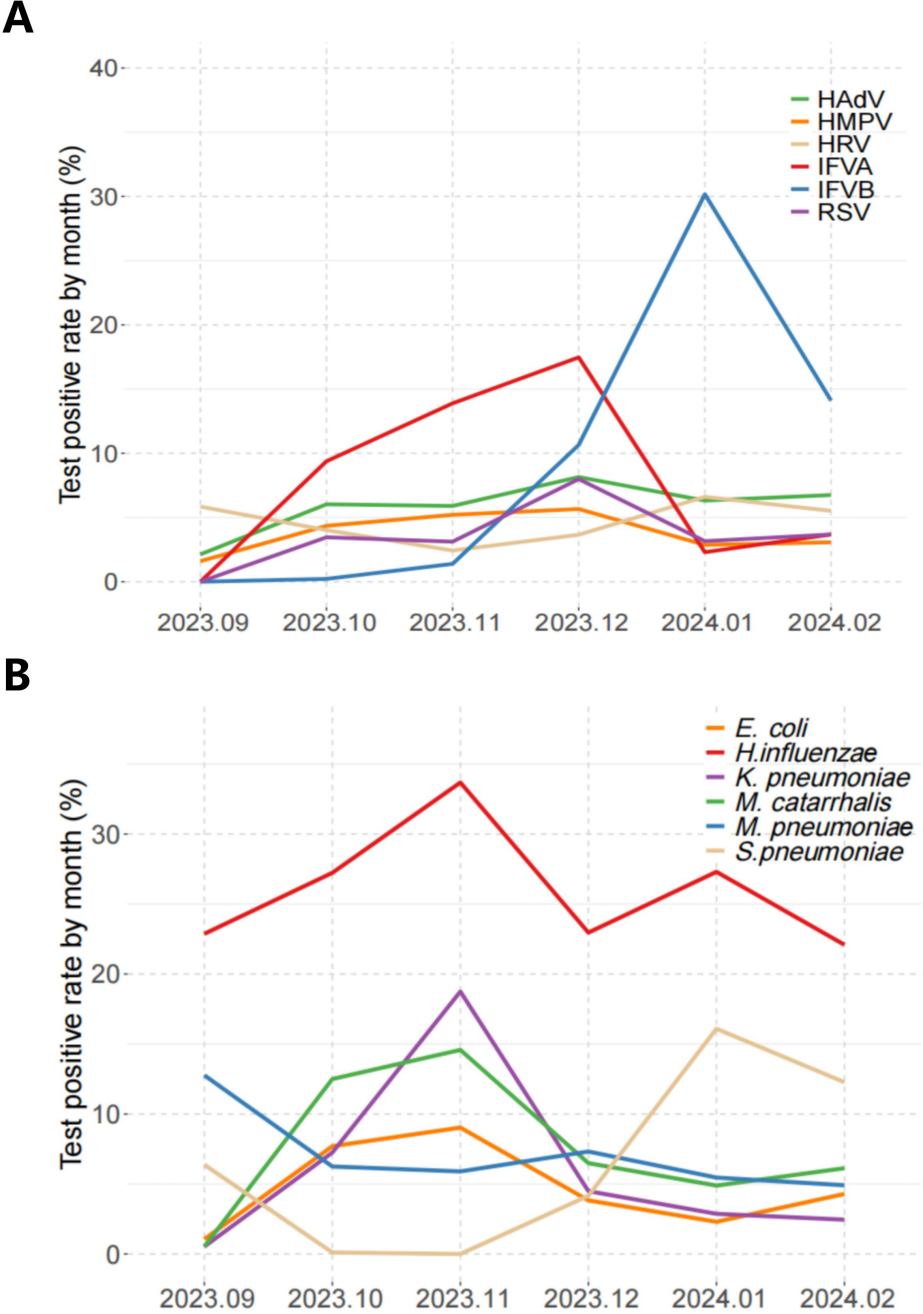
Test positive rate of pathogens by months. **(A)** The positive rate of virus tests for each month. **(B)** The positive rate of bacterial tests for each month.

Pathogen-specific temporal patterns demonstrated distinct epidemiological trajectories. IFVA, HAdV, RSV, and HMPV exhibited progressive increases from September baseline levels, while HRV maintained stable detection rates throughout the surveillance period. Notably, IFVA detection peaked in December 2023 (17.46%) before sharply declining to 2.3% by January 2024. Conversely, IFVB showed an inverse pattern, rising from December 2023 to peak at 30.17% in January 2024 (**Figure 4A**). Among bacterial pathogens, *H. influenzae* remained persistently elevated across all surveillance months. *M. catarrhalis*, *K. pneumoniae*, and *E. coli* showed transient elevations during October-November before declining to baseline levels from December onward. In contrast, *S. pneumoniae* detection rates increased substantially during December compared to preceding months (**Figure 4B**).

### Detection of multiple cytokines

**Figure 5:**
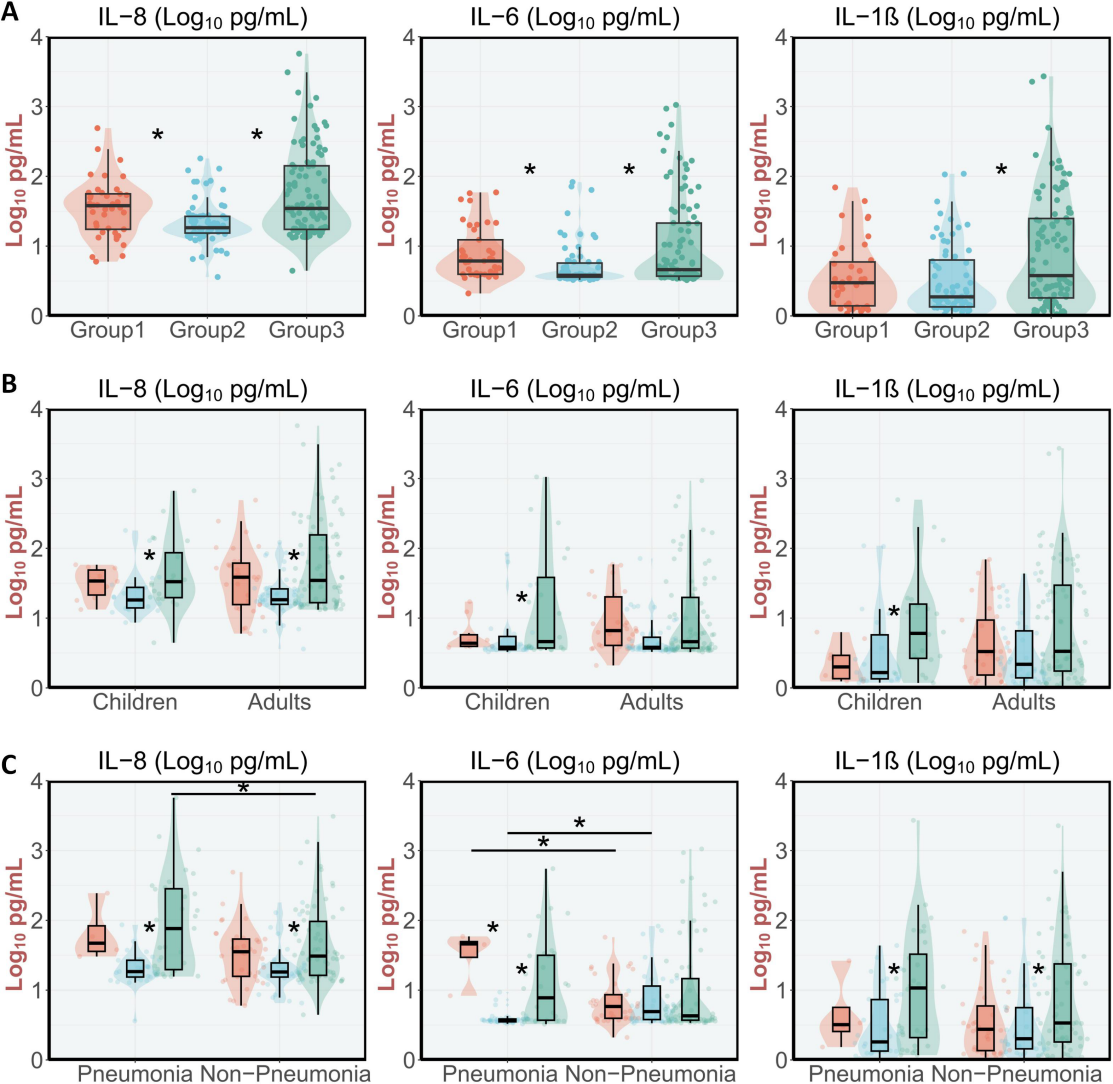
IL-8, IL-6, and IL-1β comparison of NPS of patients with ARIs and pneumonia in Beijing in one period of pre-pandemic and two periods of post-pandemic. **(A)** Cytokine concentrations in different stages. **(B)** Cytokine concentrations in pediatric and adult patients. **(C)** Cytokine concentrations in patients with pneumonia and non-pneumonia.

To characterize post-pandemic immune responses to respiratory pathogens, we analyzed cytokine profiles in nasopharyngeal swab (NPS) samples across three epidemiological cohorts. Multiplex cytokine analysis revealed significant intergroup variations specifically in IL-6, IL-8, and IL-1β levels. Group 2 demonstrated markedly reduced cytokine concentrations compared to both Group 1 and Group 3, particularly evident in IL-6 (Group 2: 3.82 [3.59-5.73] pg/mL vs Group 1: 6.12 [3.95-12.32] pg/mL vs Group 3: 4.60 [3.71-21.40] pg/mL) and IL-8 levels (Group 2: 18.35 [15.38-26.68] pg/mL vs Group 1: 37.98 [17.41-56.11] pg/mL vs Group 3: 34.7 [17.38-141.48] pg/mL) (**Figure 5A**). Subgroup analysis revealed more pronounced cytokine level alterations in pediatric patients compared to adults across all study groups (**Figure 5B**). Similarly, pneumonia patients exhibited enhanced cytokine responses relative to non-pneumonia cases, mirroring the age-related response patterns (**Figure 5C**).

## Discussion

This study examines shifts in viral, bacterial, and atypical pathogen distribution following the COVID-19 pandemic, with focus on etiological characteristics and immune system responses. Post-pandemic viral infection incidence reached 40.42%, exceeding pre-pandemic rates of 29.8%-39.3% (Yu et al., 2018; Haixu et al., 2020; Li et al., 2021), consistent with data from September-November 2023 (Gong et al., 2024). In contrast, pneumonia incidence declined to 14.98%, below the pre-pandemic rate of 33.87% (Li et al., 2021).IFVA, IFVB, HAdV, HRV, HMPV, and RSV were most frequently identified. IFVA and IFVB showed particularly elevated prevalence across adult and pediatric populations, in both pneumonia and non-pneumonia cases, surpassing pre-pandemic levels (Li et al., 2021). HAdV and RSV remained substantial contributors to childhood infections. Unexpectedly, HRV and HMPV, traditionally associated with upper respiratory infections, emerged as predominant pneumonia pathogens, exceeding IFV prevalence. HPIV4 showed increased prevalence in pediatric pneumonia cases, reflecting its stronger association with lower respiratory involvement compared to other HPIV types (Oh et al., 2021).These findings indicate an altered epidemiological landscape in the post-pandemic era, suggesting the COVID-19 pandemic modified population susceptibility or transmission dynamics of respiratory pathogens.

Epidemiological evidence indicates positive associations among *M. catarrhalis*, *H. influenzae* and *S. pneumoniae*, likely mediated by shared ecological niches, crowding conditions, and concurrent respiratory viral infections (van den Bergh et al., 2012; de Steenhuijsen Piters et al., 2015). During viral respiratory infections in children, nasopharyngeal bacterial colonization density increases significantly, potentially exacerbating ARI (Howard et al., 2019). *H. influenzae* detection exceeded 30% in our cohort, substantially higher than reported colonization rates (~17% in general populations and ~21% in healthy Chinese children) (Yang et al., 2019) (Ma et al., 2023).Conversely, *S. pneumoniae* showed an inverse age pattern: lower detection in children contrasting with previous Chinese data (21.4% (95% CI: 18.3–24.4%)) (Marking et al., 2025).COVID-19 has profoundly altered the nasopharyngeal microbiome, inducing dysbiosis associated with increased susceptibility to secondary infections and altered disease severity (Ren et al., 2021). *M. pneumoniae*, which exhibits epidemic cycles of 1–3 years (Beeton et al., 2020), showed increased detection in children during the post-pandemic period, particularly in school-age groups. This increase likely reflects resumed social activities and school attendance following pandemic restrictions. Co-infections of *H. influenzae* with respiratory viruses and *M. pneumoniae* with viruses were more prevalent, suggesting microbial community imbalance may facilitate bacterial-viral synergy. Our study reveals the complexity of nasopharyngeal microbial interactions in respiratory tract infections, demonstrating pathogen co-occurrence patterns and their clinical implications. Future investigation should elucidate mechanistic drivers of bacterial-viral pathogen synergy to inform therapeutic strategies.

During the COVID-19 pandemic, NPIs—including mask-wearing and social distancing—effectively reduced viral transmission but simultaneously diminished population-level exposure to other pathogens, thereby attenuating the “training” of adaptive immune responses (Flaxman et al., 2020; Lai et al., 2020; Brauner et al., 2021). The upper respiratory tract (URT), as the primary interface for pathogen encounter, relies on robust mucosal immune responses for early control of respiratory infections (Zhou et al., 2025). Several cytokines reflect local mucosal immune responses rather than systemic responses (Smith et al., 2021; Roubidoux et al., 2023). Most notably, IL-6 and IL-8 levels decreased significantly in 2023 compared to the pre-pandemic period (before 2020) and 2024, likely reflecting adaptive immune system remodeling following pandemic-related immune suppression. However, comorbidities (Farheen et al., 2021), vaccination status, and prior SARS-CoV-2 exposure (Padilla-Bórquez et al., 2024) can also modulate cytokine production. Hence, the suppression of the cytokines may be influenced by these potential cofounders. Cytokine profiles differed substantially between children and adults. Children demonstrated higher mucosal cytokine levels in the post-pandemic period, consistent with more robust immune activation. This elevated response likely reflects their relatively naive immune systems and ongoing immune maturation, in contrast to the more regulated responses observed in adults. Understanding temporal and age-specific variations in mucosal immune responses is essential for developing targeted therapeutic interventions and optimizing patient outcomes in respiratory tract infections.

The primary limitations encompass single-center study design, selection bias, inadequate adjustment for confounding factors, small sample sizes in cytokine analysis, lack of temporal comparability between pre- and post-pandemic cohorts, and incomplete clinical data. These factors constrain the generalizability of findings and limit causal inference capacity, necessitating improvements in future research.

In summary, this study reveals distinct patterns in respiratory pathogen epidemiology and immune responses across pediatric and adult populations in the post-pandemic period. Cytokine alterations identified in our analysis provide mechanistic explanations for age-dependent differences in infection rates and severity. These findings underscore the need for age-stratified surveillance systems and targeted therapeutic strategies to optimize respiratory infection prevention and treatment across diverse populations.

## Data Availability

The raw data supporting the conclusions of this article will be made available by the authors, without undue reservation.
