# Supplementary material for: Epidemiological and immunological insights into respiratory infections in post-COVID-19
Source: Front Cell Infect Microbiol. 2026 Jan 5;15:1634415. doi: 10.3389/fcimb.2025.1634415 (PMC12812925; doi:10.3389/fcimb.2025.1634415)
Supplement: Supplementary file 1 [file Table1.docx]

| **Aim** | **Group 1(No.)**  **(November, 2018 to December, 2019)** | **Group 2(No.)**  **(September, 2023 to February, 2024)** | **Group 3(No.)**  **(September, 2024 to December, 2024)** |
| --- | --- | --- | --- |
| **Etiological and epidemiological study** | NA | 2484 | NA |
| **Cytokines study** | 41 | 70 | 98 |

**Table S1. Cases involved in this study for different aims**

**Table S2. Characteristics of patients participating in this study**

|  |  | **Cases number** | **Percentage(%)** |
| --- | --- | --- | --- |
|  | **All** |  |  |
| **Gender** | **Male** | 1195 | 48.11 |
|  | **Female** | 1289 | 51.89 |
| **Age**  **(years)** | **Children** | **1715** | **69.04** |
|  | **<1** | 153 | 6.16 |
|  | **1-3** | 219 | 8.82 |
|  | **4-6** | 431 | 17.35 |
|  | **7-12** | 761 | 30.64 |
|  | **13-18** | 151 | 6.08 |
|  | **Adults** | **769** | **30.96** |
|  | **19-35** | 286 | 11.51 |
|  | **36-60** | 337 | 13.57 |
|  | **>60** | 146 | 5.88 |
| **Months** | **September, 2023** | 188 | 7.57 |
|  | **October, 2023** | 896 | 36.07 |
|  | **November, 2023** | 288 | 11.59 |
|  | **December, 2023** | 601 | 24.19 |
|  | **January, 2024** | 348 | 14.01 |
|  | **February, 2024** | 163 | 6.56 |

**Table S3. Positive rates of viral and bacterial and atypical infection among patients with ARI and Pneumonia**

|  |  | **Viruses tested** | | **Bacteria and atypical pathogens tested** | |  |  |
| --- | --- | --- | --- | --- | --- | --- | --- |
|  |  | **TPR (%)** | **Pneumonia (%)** | **TPR (%)** | **Pneumonia (%)** | **P value for TPR** | **P value for Pneumonia** |
|  | **All** | 40.42(1004/2484) | 33.87(126/372) | 51.45(1278/2484) | 52.15(194/372) | P＜0.05 | P＜0.05 |
| **Age(years old)** | **Children** | 41.57(713/1715) | 36.76(68/185) | 57.38(984/1715) | 68.65(127/185) | P＜0.05 | P＜0.05 |
|  | **<1** | 38.56(59/153) | 42.86(6/14) | 57.52(88/153) | 64.29(9/14) | P＜0.05 | P＞0.05 |
|  | **1-3** | 42.92(94/219) | 66.67(10/15) | 51.14(112/219) | 60.00(9/15) | P＞0.05 | P＞0.05 |
|  | **4-6** | 40.60(175/431) | 28.95(11/38) | 56.84(245/431) | 57.89(22/38) | P＜0.05 | P＜0.05 |
|  | **7-12** | 41.26(314/761) | 34.44(31/90) | 59.79(455/761) | 80.00(72/90) | P＜0.05 | P＜0.05 |
|  | **13-18** | 47.02(71/151) | 35.71(10/28) | 55.63(84/151) | 53.57(15/28) | P＞0.05 | P＞0.05 |
|  | **Adults** | 37.84(291/769) | 31.02(58/187) | 38.23(294/769) | 35.83(67/187) | P＞0.05 | P＞0.05 |
|  | **19-35** | 44.06(126/286) | 22.86(8/35) | 33.22(95/286) | 31.43(11/35) | P＜0.05 | P＞0.05 |
|  | **36-60** | 38.28(129/337) | 41.33(31/75) | 42.43(143/337) | 37.33(28/75) | P＞0.05 | P＞0.05 |
|  | **>60** | 24.66(36/146) | 24.68(19/77) | 38.36(56/146) | 36.36(28/77) | P＜0.05 | P＞0.05 |

TPR: test positive rate

*p*-values were calculated by Mann-Whitney U-test and χ2 test.

| **Table S4. Pathogen spectrum of patients with Pneumonia** | | | | | | | | |
| --- | --- | --- | --- | --- | --- | --- | --- | --- |
| **Pathogens** | **Total** | **Adults** | | | **Children** | | | **P value** |
|  |  | **Negative** | **Positive** | Percentage**（**%**）** | **Negative** | **Positive** | Percentage**（**%**）** |  |
| **Viruses** |  |  |  |  |  |  |  |  |
| HRV | 24 | 182 | 5 | 2.67 | 166 | 19 | 10.27 | P＜0.05 |
| IFVB | 23 | 168 | 19 | 10.16 | 181 | 4 | 2.16 | P＜0.05 |
| IFVA | 22 | 173 | 14 | 7.49 | 177 | 8 | 4.32 | P＞0.05 |
| HMPV | 22 | 175 | 12 | 6.42 | 175 | 10 | 5.41 | P＞0.05 |
| HAdV | 11 | 186 | 1 | 0.53 | 175 | 10 | 5.41 | P＜0.05 |
| RSV | 9 | 185 | 2 | 1.07 | 178 | 7 | 3.78 | P＞0.05 |
| PIV3 | 5 | 184 | 3 | 1.6 | 183 | 2 | 1.08 | P＞0.05 |
| PIV4 | 5 | 187 | 0 | 0 | 180 | 5 | 2.7 | P＜0.05 |
| HCoV229E | 4 | 184 | 3 | 1.6 | 184 | 1 | 0.54 | P＞0.05 |
| HCoVHKU1 | 3 | 185 | 2 | 1.07 | 184 | 1 | 0.54 | P＞0.05 |
| HBoV | 2 | 187 | 0 | 0 | 183 | 2 | 1.08 | P＞0.05 |
| PIV1 | 1 | 187 | 0 | 0 | 184 | 1 | 0.54 | P＞0.05 |
| HCoVOC43 | 1 | 187 | 0 | 0 | 184 | 1 | 0.54 | P＞0.05 |
| MeV | 0 | 187 | 0 | 0 | 185 | 0 | 0 | NA |
| PIV2 | 0 | 187 | 0 | 0 | 185 | 0 | 0 | NA |
| HCoVNL63 | 0 | 187 | 0 | 0 | 185 | 0 | 0 | NA |
| **Bacteria** |  |  |  |  |  |  |  |  |
| *H. influenzae* | 73 | 164 | 23 | 12.3 | 135 | 50 | 27.03 | P＜0.05 |
| *S. pneumoniae* | 20 | 175 | 12 | 6.42 | 177 | 8 | 4.32 | P＞0.05 |
| *E. coli* | 19 | 174 | 13 | 6.95 | 179 | 6 | 3.24 | P＞0.05 |
| *P. aeruginosa* | 18 | 178 | 9 | 4.81 | 176 | 9 | 4.86 | P＞0.05 |
| *M. catarrhalis* | 17 | 184 | 3 | 1.6 | 171 | 14 | 7.57 | P＜0.05 |
| *K. pneumoniae* | 13 | 181 | 6 | 3.21 | 178 | 7 | 3.78 | P＞0.05 |
| *S. aureus* | 11 | 181 | 6 | 3.21 | 180 | 5 | 2.7 | P＞0.05 |
| *A. baumannii* | 9 | 182 | 5 | 2.67 | 181 | 4 | 2.16 | P＞0.05 |
| *B. pertussis* | 7 | 187 | 0 | 0 | 178 | 7 | 3.78 | P＜0.05 |
| GAS | 0 | 187 | 0 | 0 | 185 | 0 | 0 | NA |
| *L. pneumophila* | 0 | 187 | 0 | 0 | 185 | 0 | 0 | NA |
| **Atypical pathogens** |  |  |  |  |  |  |  |  |
| *M. pneumoniae* | 69 | 180 | 7 | 3.74 | 123 | 62 | 33.51 | P＜0.05 |
| *C.pneumoniae* | 0 | 187 | 0 | 0 | 185 | 0 | 0 | NA |

*p*-values were calculated by Mann-Whitney U-test and χ2 test.

**Table S5. Positive rates of patients with single and multiple infection**

|  | **Group** | **Single infection(%)** | **P value** | **Muitiple infection(%)** | **P value** |
| --- | --- | --- | --- | --- | --- |
|  | **All** | 38.85(965/2484) |  | 31.88(792/2484) | P＜0.05 |
| **Gender** | **Male** | 37.32（446/1195） | P>0.05 | 35.15（420/1195） | P＜0.05 |
|  | **Female** | 40.26（519/1289） |  | 28.86（372/1289） |  |
| **Age（years old）** | **Children** | 36.85（632/1715） | P＜0.05 | 37.26(639/1715） | P＜0.05 |
|  | **<1** | 36.60（56/153） |  | 35.29(54/153) |  |
|  | **1-3** | 31.96(70/219) |  | 36.53(80/219) |  |
|  | **4-6** | 35.27(152/431) |  | 38.05(164/431) |  |
|  | **7-12** | 38.90(296/761) |  | 37.71(287/761) |  |
|  | **13-18** | 38.41(58/151) |  | 35.76(54/151) |  |
|  | **Adults** | 43.30(333/769） |  | 19.90(153/769） |  |
|  | **19-35** | 48.95(140/286) |  | 16.43(47/286) |  |
|  | **36-60** | 42.14(142/337) |  | 23.74(80/337) |  |
|  | **>60** | 34.93(51/146) |  | 17.81(26/146) |  |
| **Month** | **September, 2023** | 36.7(69/188） | P>0.05 | 18.09(34/188) | P＜0.05 |
|  | **October,2023** | 38.5(345/896） |  | 31.81(285/896） |  |
|  | **November,2023** | 37.15(107/288） |  | 38.54(111/288) |  |
|  | **December,2023** | 41.10(247/601） |  | 32.78(197/601) |  |
|  | **January,2024** | 40.23(140/348） |  | 33.91(118/348) |  |
|  | **February,2024** | 34.97(57/163） |  | 28.83(47/163) |  |

*p*-values were calculated by Mann-Whitney U-test and χ2 test.
